# The ethical responsibility to continue investigational treatments of research participants in situation of armed conflicts, economic sanctions or natural catastrophes

**DOI:** 10.3389/fmed.2022.950409

**Published:** 2022-08-09

**Authors:** Sandor Kerpel-Fronius, Chieko Kurihara, Francis P. Crawley, Varvara Baroutsou, Sander Becker, Brigitte Franke-Bray, Kotone Matsuyama, Shehla Naseem, Johanna Schenk

**Affiliations:** ^1^Department of Pharmacology and Pharmacotherapy, Semmelweis University, Budapest, Hungary; ^2^Kanagawa Dental University, Kanagawa, Japan; ^3^Good Clinical Practice Alliance—Europe (GCPA) & Strategic Initiative for Developing Capacity in Ethical Review, Leuven, Belgium; ^4^Consultant, Pharmaceutical Medicine, Athens, Greece; ^5^Consultant in Pharmaceutical Medicine, Dover Heights, NSW, Australia; ^6^Independent Consultant, Basel, Switzerland; ^7^Department of Health Policy and Management, Nippon Medical School, Tokyo, Japan; ^8^Ferozsons Laboratories Ltd, Karachi, Pakistan; ^9^PPH plus GmbH & Co. KG, Hochheim am Main, Germany

**Keywords:** Ukraine war, economic sanctions, vulnerable population, clinical trials, ethics, investigational treatment

## Abstract

This paper discusses the effects of armed conflict, economic sanctions, and natural catastrophes on ongoing clinical trials. We suggest that

• stopping the accrual of new patients in clinical trials under such extreme conditions is acceptable.

• research participants already receiving trial medication in such disruptive situations are to be considered highly vulnerable due to their medical dependency for ongoing treatment according to the approved clinical study protocol.

• based on the present experience in Ukraine and Russia, we conclude that finishing ongoing trial treatments according to approved or amended protocols should be considered to be an ethical obligation of trial sponsors irrespective whether trial disruption is due to war, economic sanctions, or natural catastrophes.

• it is important to devote more attention to the ethical challenges raised by such fundamentally disruptive situations to clinical trials generally in any region of the world.

The Russian attack on Ukraine initiated a cruel and protracted war leading to an extensive death toll of the local population and to the broad destruction of the industrial, social and medical infrastructure in Ukraine. As a response to the military aggression, Western countries organized wide international economic sanctions against Russia with the aim of crippling the Russian economy and causing much discomfort across the society. The hope was that these measures would weaken the Russian economic backbone needed to continue the war while also persuading the Russian people and authorities to end the war. Indeed we can speak now of two types of wars running parallel. The military war causes extensive fatalities along with the destruction of the infrastructure in Ukraine while the sanctions affect only the economic life of Russia and its citizens, causing significant disruption and suffering primarily for the poorer segment of the population. Both of these countries were important hubs for international clinical trials.

The Ethics Working Group of the International Federation of Associations of Pharmaceutical Physicians & Pharmaceutical Medicine (IFAPP) considers it important that clinical studies take into account the ethical responsibility of continuing ongoing investigational treatments according to the protocol in patients already enrolled in trials in both countries. Seriously ill patients already involved in clinical trials are highly vulnerable due to the fact that in some cases the initiated investigational treatment might be their last therapeutic option or it may be the best medically advised pathway. Research participants should not be summarily abandoned during either type of warfare irrespective to which warrior parties the patients belong (1). Below, we shall shortly review the clinical trial scene in these countries and discuss the background of our ethical recommendation while going on to analyze the actions taken by companies performing trials in these countries.

The interests of pharmaceutical companies have in running clinical trials in both Ukraine and Russia are complex and quite similar. Both countries have a large, often not pretreated, patient population. The participation in clinical trials is frequently the only possibility for both the patients and the medical team to get access to breakthrough, innovative drugs. In addition, such trials secure much needed financial income for the participating institutes. Only a limited number of well equipped leading clinical centers are able to participate in industry-sponsored trials. Though the war lies at the basis of the current situation, the causes for the severe disruption of clinical trial activities are very different in the two countries (2).

The number of ongoing clinical trials in these countries cannot be accurately determined. In Ukraine there appeared to be around 700 ongoing or planned trials and around 60% still recruiting participants when the war started. The majority of the studies were in oncology, most of which focused on lung cancer. Other important patient groups included those in the cardiovascular, respiratory, gastro-intestinal and infectious diseases (3). In Ukraine the war has thus far caused severe damages in the infrastructure, destroyed several hospitals, killed many inhabitants including both patients and healthcare personnel. In addition, a large fraction of the population emigrated from the war-stricken regions to the western region of Ukraine or to other countries. These changes made clinical trial work practically impossible in the regions where there was heavy fighting and much destruction. Many enrolled patients were lost from the trials. Only a few could continue treatments in hospitals located in western Ukraine or in the neighboring countries. Moreover, the investigational medicines supply to local centers as well as the sending of trial samples and data of patients became impossible or unreliable (2, 4, 5).

In Russia around 600 foreign sponsored trials are ongoing, most of them are large studies sponsored by international companies. Similar to the situation in Ukraine the overwhelming majority of these studies accrued cancer patients. The greater majority of the studies are phase 3 clinical trials, while phase 2 and phase 1–2 studies represent a much lower fraction. The Russian centers are operational; the infrastructure of the country is intact. Neither the hospital staff nor the patients are threatened or displaced (6). The international trials were brought to halt by economic sanctions designed to cripple economic life and consequently weakening Russia' ability to continue the war. All pharmaceutical companies were requested to end their engagement in Russia including the running of clinical trials in Russia. Dr. Caplan, a wellknown ethicist in the US provided an ethical backing for this policy: he argued that both medicine and science are politically controlled. Accordingly, pharmaceutical companies should support political efforts to undercut the economic basis of inhumane politics leading to war crimes and killing many thousands of civilians. However, he pointed out that the harm to existing subjects of treatment should be minimized over a short transitional time (7).

Most of the leading drug companies complied with this policy, although stopping business in healthcare might lead to several ethical problems (8). The economic sanctions have at the time of this writing disrupted only a small number of studies entirely. Russian investigators stated that they have some study medication stockpiled locally for continuing the treatment of patients already in trials. In addition, several companies have explicitly stated that they will continue supplying drugs for already initiated trial treatments as described in the protocols. However, sooner or later stopping the accrual of new patients and the depletion of the existing drug supplies will bring international studies to a full stop cutting off Russian patients and clinical researchers from participating in international clinical research. Moreover, the cancellation of flights between Russia and Western countries effectively interrupts the transport of biomaterials to international laboratories making the combined evaluation of treatment effects scientifically questionable. Russian sponsored local trials do not appear to be affected (9).

Many ethics questions emerge in connection with ongoing clinical trials in time of armed conflict, economic sanctions, and natural disasters. The decision as to where to place a trial and when to stop entering new patients into a study is a combined scientific and strategic decision for sponsors. Accordingly, the cessation of trial enrollment as part of economic sanctions can be considered ethically acceptable. However, after starting an investigational treatment of a trial patient the sponsor enters into a medical ethical obligation to perform the therapy according to the study protocol to the best of the sponsor's ability. Stopping treatment might deprive seriously ill patients from possible effective, perhaps life-saving, experimental therapies. The rapid withdrawal of treatment may even endanger the trial participants. Therefore, from an ethical perspective, we have to consider patients already enrolled in trials in time of war as belonging to a highly vulnerable population. They need specific protection irrespective of whether their safety is endangered by military or economic warfare and without consideration to which warring parties they may belong. Importantly, the medical neutrality of healthcare professionals must be maintained (10).

The ethical requirement for continued investigational treatment of trial participants in time of conflicts is a newly surfacing challenge during the war in Ukraine. With the rapidly growing number of large trials accruing patients from many countries, the many smaller local wars frequently accompanied by economic sanctions will unfortunately affect an increasing number of enrolled subjects in the future. Although this patient group is small compared to the large number of people whose health or healthcare is endangered by military and economic actions, it represents a special challenge in ethics for pharmaceutical physicians, clinical pharmacologists, investigators, sponsors, members of ethics committees, regulators and patient organizations; in short for all those involved in regulating, organizing and evaluating clinical research.

The dilemma of study participants trapped in war can be related to humanitarian laws, which have evolved to ensure ongoing human rights frameworks in time of war and other fundamental disruptions to society and the rule of law (10, 11). The Geneva Convention, the basis of humanitarian laws aims, to protect combatants who are put out of action through sickness, injury, or having become prisoners of war. It also deals with persons not directly participating in hostilities while prohibiting the destruction of healthcare facilities (12). In the UNESCO Universal Declaration on Bioethics and Human Rights, the ethical principles governing research on human subjects were firmly connected to rules governing respect for human dignity and human rights (13). Furthermore, the ethical issues of clinical studies carried out during war or other catastrophes became part of the International Ethical Guidelines for Health-related Research Involving Humans [Council for International Organizations of Medical Sciences (CIOMS)]. In this document, the vulnerability of patients was redefined as “*the judgment that groups are vulnerable is context dependent and requires empirical evidence to document the need for special protections”* (14). We argue here that in the context of war and dependence on treatments provided through clinical trials require us to view research participants in conflict (and other fundamentally disruptive situations) as particularly vulnerable.

We postulate that patients already enrolled into clinical trials become vulnerable when, due to any type of disaster, the continuation of their therapy with investigational drugs becomes problematic. Above we showed that this is now the situation in Ukraine due to destruction of the social and healthcare infrastructures. In Russia the problem arises due to the economic sanctions, which also significantly interfere with the normal functioning of healthcare and disrupt the drug supply intended for clinical studies. The specific clinical vulnerability of the patients already on study treatment led us to formulate the appeal of the IFAPP Ethics Working Group presented in the introductory paragraph (1). Most especially we wish to call the attention of clinical trial organizers that extensive economic warfare broadly affecting social functions can also significantly disrupt healthcare activities and completely inhibit the conduct of clinical trials even where human lives and hospitals are not directly targeted. This is exactly the goal of economic warfare that explicitly includes the role of pharmaceutical companies (7). Therefore, even under situations of severe economic sanctions the ethical norm of medical neutrality and special care for vulnerable persons must be maintained, including the realm of clinical research.

According to their own statements the pharmaceutical companies have tried to maintain their ongoing trials in Ukraine mostly by continuing treatments in quieter regions in Ukraine or in other European countries. The European Medicines Agency (EMA) has helped by providing notification on how to handle trial records, protocol deviations, missing data, and other areas of concern (15). We also analyzed the statements of 10 western pharmaceutical companies that have organized the most of the international studies in Russia (9). All of these companies pledged to follow the economic sanctions, limit their commercial actions to lifesaving drugs and stop the accrual of patients into ongoing trials (16). Some companies, including Merck (17), Sanofi (18), and Pfizer (19) explicitly stated that they would continue to provide drugs for treating patients already enrolled into trials.

This tragic war in Ukraine and the reactive economic sanctions against Russia interfere significantly with ongoing international clinical trials. This is without precedence in history. Almost 3 months after the start of this armed and economic warfare, we find that several international drug companies have significantly reflected on the ethics dimensions of this dramatic situation for clinical research. They have done their best to continue to support their trial participants, investigators, and staff in the combat areas, guided primarily by ethics considerations and the requirements for secure clinical research. Although the war and the economic sanctions have significantly interrupted broad drug trade and trial activities, many companies decided to minimize the clinical and ethical damage by maintaining protocol-defined treatment for patients already enrolled in clinical trials. Based on this experience, we conclude that several clinical trial sponsors have provided significant consideration to their ethical obligations and continue to look for the best solution for their trial participants and the trials themselves during the war.

There are no adequate data available as yet on just how well the clinical research community has been able to secure the continuation of study medications in this war. However, we need to learn from this war for future wars and natural catastrophes, which might cause also devastating damage to society and its infrastructure. It is important now that more attention is brought to the ethical challenges raised by such fundamentally disruptive situations to clinical trials generally in a region.

## Author contributions

SK-F, CK, and FC: substantial contributions to the conception or design of the work, or the acquisition, analysis or interpretation of data for the work, and revising it critically for important intellectual content. VB, SB, BF-B, KM, SN, and JS: drafting the work or revising it critically for important intellectual content. All authors contributed to the article and approved the submitted version.

## Conflict of interest

Author SN was employed by Ferozsons Laboratories Ltd and JS was employed by PPH plus GmbH & Co. KG. The remaining authors declare that the research was conducted in the absence of any commercial or financial relationships that could be construed as a potential conflict of interest.

## Publisher's note

All claims expressed in this article are solely those of the authors and do not necessarily represent those of their affiliated organizations, or those of the publisher, the editors and the reviewers. Any product that may be evaluated in this article, or claim that may be made by its manufacturer, is not guaranteed or endorsed by the publisher.
